# Creating a clinical platform for carbon‐13 studies using the sodium‐23 and proton resonances

**DOI:** 10.1002/mrm.28238

**Published:** 2020-03-13

**Authors:** James T. Grist, Esben S.S. Hansen, Juan D. Sánchez‐Heredia, Mary A. McLean, Rasmus Tougaard, Frank Riemer, Rolf F. Schulte, Joshua D. Kaggie, Jan Henrik Ardenkjaer‐Larsen, Christoffer Laustsen, Ferdia A. Gallagher

**Affiliations:** ^1^ Department of Radiology University of Cambridge Cambridge United Kingdom; ^2^ Institute of Cancer and Genomic Sciences University of Birmingham Birmingham United Kingdom; ^3^ MR Research Centre Aarhus University Aarhus Denmark; ^4^ Department of Health Technology Technical University of Denmark Kgs. Lyngby Denmark; ^5^ CRUK Cambridge Institute Cambridge United Kingdom; ^6^ GE Healthcare Munich Germany; ^7^ GE Healthcare Chicago USA

**Keywords:** calibration, carbon‐13, hyperpolarized, MRI, sodium‐23

## Abstract

**Purpose:**

Calibration of hyperpolarized ^13^C‐MRI is limited by the low signal from endogenous carbon‐containing molecules and consequently requires ^13^C‐enriched external phantoms. This study investigated the feasibility of using either ^23^Na‐MRI or ^1^H‐MRI to calibrate the ^13^C excitation.

**Methods:**

Commercial ^13^C‐coils were used to estimate the transmit gain and center frequency for ^13^C and ^23^Na resonances. Simulations of the transmit *B*_1_ profile of a Helmholtz loop were performed. Noise correlation was measured for both nuclei. A retrospective analysis of human data assessing the use of the ^1^H resonance to predict [1‐^13^C]pyruvate center frequency was also performed. In vivo experiments were undertaken in the lower limbs of 6 pigs following injection of hyperpolarized ^13^C‐pyruvate.

**Results:**

The difference in center frequencies and transmit gain between tissue ^23^Na and [1‐^13^C]pyruvate was reproducible, with a mean scale factor of 1.05179 ± 0.00001 and 10.4 ± 0.2 dB, respectively. Utilizing the ^1^H water peak, it was possible to retrospectively predict the ^13^C‐pyruvate center frequency with a standard deviation of only 11 Hz sufficient for spectral–spatial excitation‐based studies.

**Conclusion:**

We demonstrate the feasibility of using the ^23^Na and ^1^H resonances to calibrate the ^13^C transmit *B*_1_ using commercially available ^13^C‐coils. The method provides a simple approach for in vivo calibration and could improve clinical workflow.

## 1 INTRODUCTION

Hyperpolarized ^13^C MRI is an emerging technique to noninvasively image cellular metabolism in health and disease: the exchange of hyperpolarized ^13^C‐pyruvate to ^13^C‐lactate is the most studied in vivo reaction using the technique.^1^, ^2^, ^3^ The recent translation of the technology into human studies has demonstrated applications for the method in prostate and brain tumors as well as the normal heart and brain.^4^, ^5^, ^6^, ^7^, ^8^ However, there are a number of challenges to be overcome before the technology can be used more routinely. For example, calibration of the RF amplifier gain (here referred to as *RF gain* as well as *transmit gain* (TG)*, coil voltage*, and *coil reference scale*), *center frequency* (*f*_0_), and estimation of transmit–receive *B*_1_ for ^13^C can be challenging given the very short time window for hyperpolarized ^13^C imaging following a single injection of hyperpolarized ^13^C labelled substrate. Calibration is usually undertaken prior to the detection of the transient hyperpolarized ^13^C‐pyruvate signal in vivo. A number of solutions to this problem have been proposed, including the use of a phantom containing a high concentration of ^13^C‐enriched molecules for calibration, as well as phantom acquisition prior to patient scanning.^9^ Although these methods provide an estimation of RF gain for the field of view (FOV) containing the phantom, there is no current method to establish the transmit *B*_1_ field over the whole FOV, which is particularly relevant because variations in the delivered flip angle are expected throughout the patient. Furthermore, the position and composition of the phantom increase the uncertainty of centre frequency estimation.

A selective excitation approach is frequently employed to acquire high signal to noise ratio images of the metabolic products of [1‐^13^C]pyruvate. Deviations in the flip angle across the FOV can introduce significant uncertainty in the kinetic parameters that are derived using this spectral–spatial imaging approach.^10^, ^11^ A further challenge is the estimation of coil sensitivity maps, which is used to accurately combine the images from individual coils. This absence of significant signal from natural abundance ^13^C renders these challenges difficult to solve without the use of a phantom. An interesting calibrationless approach has been reported that uses the pyruvate signal to estimate the sensitivity map for in vivo studies.^12^

Here, we propose using the endogenous signal derived from the ^1^H and ^23^Na nuclei as a reference for the ^13^C imaging: ^23^Na has a resonant frequency similar to that of ^13^C at clinical field strengths (~1 MHz difference at 3 tesla) with a relatively high natural abundance in the body, producing the second highest signal on MRI from the nuclei detectable in biological tissues. The ^1^H nucleus is the highest natural abundance NMR active signal in the body and thus represents a further option to confirm center frequency placement prior to ^13^C acquisition. A number of clinical studies have demonstrated the biodistribution of sodium using ^23^Na‐MRI in both health and disease.^13^, ^14^, ^15^, ^16^ Previous reports have utilized a variable capacitor custom‐built RF coil to enable the coil to be tuned to both the ^13^C and ^23^Na frequency, allowing accurate RF gain estimation for rodent studies.^17^, ^18^ In this study, we have expanded on this work to study the use of commercially available single tuned ^13^C coils to acquire transmit *B*_1_ and *f*_0_ information from the ^23^Na resonance using a clinical system in large animals, therefore providing the translational leap required for this technique to be incorporated into routine clinical practice. Furthermore, we have incorporated the use of the ^1^H nucleus to provide further confidence in the estimated ^13^C center frequency derived from the ^23^Na measurements. This approach could improve the workflow of hyperpolarized ^13^C‐MRI experiments by removing the reliance on external phantoms for the prescan acquisition on clinical systems using the data acquired on commercially available coils.

## 2 METHODS

Experiments were performed at 2 different sites (MR Research Centre, University of Aarhus, Denmark; Department of Radiology, University of Cambridge, UK), which are referred to as sites A and B, respectively.

Human study: Local ethical approval was obtained for this prospective study (NRES Committee East of England, Cambridge South, REC number 15/EE/ 0255).

Porcine study: All porcine imaging was undertaken in accordance with the Danish Animal Welfare Act 2013 following an explicit national ethical review process undertaken by the Danish Animal Experiments Inspectorate.

### 2.1 Phantom and coil setup

Phantom experiments were performed at site A using a ^13^C‐labeled urea (8 M, Merck, Darmstadt, Germany) vial and identical 1 L containers filled with saline at varying concentrations (156, 117, 78, 39 mmolL^−^^1^). The ^13^C‐urea phantom was placed on top of the saline phantom and secured. The receive coils used in this study were tuned to ^13^C and included a simple loop coil (Rapid Biomedical, Rimpar, Germany) and 8‐channel paddle coils (GE Healthcare, Waukesha, WI, USA). The loop coil was placed on top of the ^13^C‐urea phantom, and the paddle coils were placed around the right and left side of the saline bottles. A separate ^13^C‐tuned clamshell volume coil (Rapid Biomedical, Q unloaded = 270, Q loaded = 75, S11 < −150 cB) was used for transmission.^19^ Data acquisition was undertaken using a 3 tesla scanner (MR750, GE Healthcare). Phantom and coil positioning can be seen in Supporting Information Figure S1.

### 2.2 RF gain and *f*_0_ estimation

Using the phantom setup described above, RF gain and *f*_0_ were assessed using a commercially available pulse‐acquire sequence (Fidall, GE Healthcare) with 2 off‐resonance Bloch‐Siegert pulses (excitation pulse width = 0.5 ms, repetition time = 2 s, echo time = 0.5 ms, flip angle = 90°).^20^ Estimation of RF gain and *f*_0_ for ^13^C and ^23^Na were performed in series. Experiments were repeated in triplicate, with the phantom removed and re‐sited between each repeat. ^1^H, ^13^C, and ^23^Na acquisitions were acquired for all saline concentrations. Scaling factors to convert between the ^1^H *f*_0_, ^23^Na *f*_0_, and the ^13^C *f*_0_ were derived from Equations 1 and 2:

(1) $${}^{23}Na,\;{}^{13}C\;Frequency\;scaling\;factor\;=\;\frac{f_{0}^{{}^{23}Na}}{f_{0}^{{}^{13}C}}$$

(2) $${}^{1}H,\;{}^{13}C\;Frequency\;scaling\;factor\;=\;\frac{f_{0}^{{}^{1}H}}{f_{0}^{{}^{13}C}},$$

where $f_{0}^{{}^{1}H}$, $f_{0}^{{}^{23}Na}$, $f_{0}^{{}^{23}Na}$, and $f_{0}^{{}^{13}C}$ are the center frequencies of ^1^H, ^23^Na, and ^13^C, respectively.

A further correction for the difference in TG between ^23^Na and ^13^C was derived using Equation 3. Because RF gain is logarithmically scaled, the subtraction rather than the ratio between ^13^C and ^23^Na RF gain is used.

(3) $$Gain\;correction\;=\;TG^{{}^{23}Na}-TG^{{}^{13}C},$$

where $TG^{{}^{23}Na}$ and $TG^{{}^{13}C}$ are the required TG for ^23^Na and ^13^C, respectively.

The mean scaling factors for frequency and gain were derived by averaging across all experiments.

### 2.3 Multinuclear transmit *B*_1_ and *B*_0_ mapping

Carbon‐13 and sodium‐23 transmit *B*_1_ maps were calculated using a cylindrical 8 L saline/pyruvate phantom (150 and 102 mmolL^−1^ respectively, doped with 80 mL Gadovist to shorten the ^13^C T_1_ below 1 s; ^1^H‐MRI image shown in Supporting Information Figure S2A), a 16‐channel receive array coil (Rapid Biomedical, Rimpar, Germany), and a clamshell transmit coil, and by employing the double angle method.^21^ Acquisition parameters were as follows: 2D‐chemical shift imaging (CSI), slice thickness = 60 mm, spectral width = 5000 Hz, spectral resolution = 256 points, FOV = 240 mm, matrix = 8 × 8, flip angles = 40 and 80, ^13^C number of averages (NEX) = 32, ^23^Na NEX = 64, ^13^C repetition time = 2.5s, ^23^Na repetition time = 600 ms, RF pulse = partially self‐refocusing sinc excitation (1.8 ms, bandwidth 2289 Hz for ^13^C) or hard pulse (pulse width = 2 ms, for ^23^Na), ^23^Na echo time = 1.2 ms, and ^13^C echo time = 1.8 ms. The width of the hard pulse is 4 times longer than the default (0.5 ms). Because ^23^Na requires approximately 12 dB more power than ^13^C, the pulse width for ^23^Na was quadrupled, although the amplitude was fixed to remain the same as for the default pulse width. Spectral data were zero‐filled in the time domain to 1024 points, Fourier‐transformed, zero‐order–phased, and fit with a Lorentzian function prior to calculation of *B*_0_ and *B*_1_ maps. All processing was performed in MatLab (2018b, MathWorks, Natick, MA).

*B*_0_ maps were calculated on a voxel‐by‐voxel basis by finding the center of the pyruvate or sodium resonance relative to the system center frequency for the inner 6 × 6 voxels of the 2D CSI grid. The mean difference in center frequency for both resonances was calculated over the phantom. The mean difference between ^13^C and ^23^Na *B*_0_ maps was calculated for the 6 × 6 grid in Hertz, as well as the mean *B*_0_ shift across the FOV for each nucleus in Hertz.

The mean *B*_1_ ratio, as well as the mean absolute percentage difference defined in Equation 4, at and between both frequencies was calculated from the inner 6 × 6 voxels of the 2D CSI grids:

(4) $$Percentage\;difference_{x,y}=100\left(\frac{B_{1\left(x,y\right)}^{{}^{13}C}-B_{1\left(x,y\right)}^{{}^{23}Na}}{B_{1\left(x,y\right)}^{{}^{23}Na}}\right),$$

where *x* and *y* are the spatial locations of each voxel in the 6 × 6 CSI grid.

### 2.4 Simulations

**FIGURE 1:**
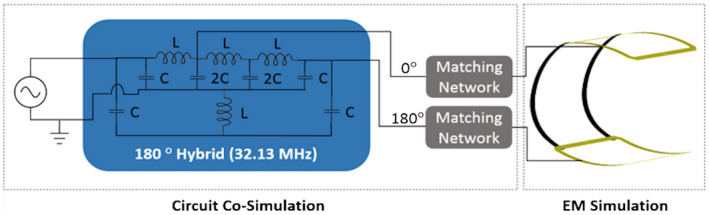
Schematic of the simulated model of the clamshell‐type transmit coil with the hybrid circuit needed to excite the homogenous mode (C = 140 pF, L = 350 nH)

The $B_{1}^{+}$ distribution of the clamshell volume coil was simulated using the frequency solver within Computer Simulation Technology software (CST, CST 2018, Darmstad, Germany). The coil consisted of 2 square loops of 300 mm diameter placed coaxially with the centers separated by 360 mm. This coil design provides a homogenous resonance mode when the signal in its 2 loops is shifted 180°. For the analysis in this study, it was important to include in the simulation a real implementation of the phase shifter used to create the homogenous mode because it is also frequency‐dependent. For that purpose, a lumped‐element 180° hybrid circuit^22^ tuned to the ^13^C frequency was included in the simulation using the circuit cosimulation feature within the software. A schematic of the whole simulation setup is shown in Figure 1, including the matching networks (lattice baluns) needed between the 180° hybrid and the loops. The transmit *B*_1_ field distributions for 1 W of accepted power were calculated as per Equation 5 and compared between the ^13^C (32.13 MHz) and ^23^Na (33.79 MHz) frequencies.

(5) $$B_{1}\;Difference\;=20\;\log\left(\frac{B_{1}^{{}^{23}Na}}{B_{1}^{{}^{13}C}}\right).$$

The results are presented in a plane orthogonal to the *B*_0_ direction and averaged over a 100 mm slice.

### 2.5 Multichannel coil noise correlation

Noise correlation was assessed using the 8‐channel paddle receive coils in conjunction with the clamshell transmit. To acquire a noise‐only scan, the RF amplifier was disabled, and multichannel receive data was acquired at both ^13^C and ^23^Na frequencies.

### 2.6 System magnetic field drift and ^1^H center frequency estimation from human studies

A retrospective analysis of previously acquired healthy (*N* = 8) and patient (*N* = 9) brain data acquired at site B between March 2016 and October 2018 was performed. Experiments were performed on a 3 T MR750 MR system (GE Healthcare) using a quadrature birdcage ^13^C/^1^H head coil (Rapid, Rimpar, Germany).

A phantom containing either approximately 8 mL of 8 M ^13^C‐urea or 1 M ^13^C‐bicarbonate or 2 mL of 4 M [1‐^13^C]lactate was placed inside a disposable cover on the superior edge of the ear defenders for use during the calibration of frequency and pulse power, then generally removed prior to the hyperpolarized pyruvate injection.

Slice‐selective ^13^C‐MR spectra and/or images were acquired from the brains of the 17 subjects following injection of 0.4 ml/kg of hyperpolarized [1‐^13^C]pyruvate, as described previously.^8^ Slice‐selection was performed using either a spectral–spatial pulse with a pass‐band of 85 Hz (22.5 ms duration, or in most cases a partially self‐refocusing sinc excitation as described above).^11^, ^23^

Individual experimental parameters varied as the protocol developed. In in the majority of subjects, however, 3 axial slices were collected (3 cm thick, 3 mm spacing) using the iterative decomposition using echo asymmetry and least squares estimation spiral CSI technique,^12^ with a cycle of 8 steps, including 1 slice‐localized spectrum and 7 single‐shot spiral images with incremented echo time from which images were reconstructed at the frequency offsets for pyruvate, lactate, bicarbonate, alanine, and pyruvate hydrate. The usual temporal resolution of the dynamic acquisitions was 4 s (sequence repetition time 500 ms), excitation flip angle 15°, image FOV 24 cm, effective true resolution in‐plane 12 mm, interpolated to 1.875 mm.

Spectra were analyzed in MatLab to measure the actual frequency of both ^13^C‐pyruvate from the central brain slice in the time‐averaged dynamic series and the reference standard during the preinjection calibration scan. The relationship was characterized between the actual ^13^C‐pyruvate frequency in vivo and 3 different parameters, which could be used as a predictor of frequency (Temporal Drift, Phantom, and Water ^1^H methods, described below). For each of these methods, a Bland‐Altman analysis was performed comparing the pyruvate frequency predicted using that method and the true measured value for all subjects to retrospectively determine which method would have performed best.

1. Temporal Drift method. The frequency of pyruvate in vivo was assumed to vary in a slow linear fashion due to drift in the system *B*_0_. The transmit frequency for a particular subject was therefore calculated from the slope of a linear fit to frequency against the date that the study was performed for all 17 subjects.
2. Phantom method. The frequency difference between pyruvate in vivo and the reference metabolite within the external phantom was tabulated, and the mean offset over all subjects was applied in each individual.
3. Water ^1^H method. The frequency of water in the ^1^H‐MRI series immediately preceding the ^13^C experiment was recorded and divided by the ratio of the gyromagnetic ratio (γ) for ^1^H/^13^C (3.97595) to estimate the ^13^C frequency. The additional offset of [1‐^13^C]pyruvate from this frequency measured in vivo was tabulated, and the mean offset determined from all the subjects was applied in each individual.

### 2.7 In vivo experiments using a ^23^Na and ^1^H prescan

A prospective study assessing the use of a ^23^Na and ^1^H prescan was performed at site A. Six female, Danish domestic pigs weighing ∼30 kg and fasted overnight were imaged. The pigs received intravenous propofol (12 mg initial dose; thereafter 0.4 mg/kg/h for maintenance anesthesia) and intravenous fentanyl (8 μg/kg/h) and were mechanically ventilated. Catheterization was performed through the femoral veins and arteries for the administration of hyperpolarized [1‐^13^C]pyruvate and measurement of arterial blood pressure, respectively.

The pigs were imaged in a supine position, and the ^13^C‐receive loop was positioned centrally on the biceps femoris muscle of the right lower limb. The receive coil and pig were placed in the central transmit field of the ^13^C‐transmit coil. Bloch‐Siegert experiments were performed prior to the ^13^C‐pyruvate injection for both the ^23^Na calibration acquisitions, with ^1^H center frequency taken from the previous series, and the ^13^C prescan acquired with ^13^C‐lactate phantom. Frequency scaling between ^1^H/^23^Na and ^1^H/^13^C pyruvate was calculated from Equations 1 and 2, respectively. Approximately 24 mL of ~250 mM hyperpolarized ^13^C‐pyruvate was injected in the left femoral vein over 10 s, followed by a 20 mL saline flush. ^13^C magnetic resonance spectroscopy was performed at the commencement of the ^13^C‐pyruvate injection using the following parameters: partially self‐refocusing sinc excitation,^23^ RF bandwidth = 2000 Hz, spectral acquisition bandwidth = 5000 Hz, number of samples = 2048, repetition time = 1 s, time points = 128, flip angle = 12°, and slice thickness = 40 mm.

### 2.8 In vivo spectral postprocessing

Hyperpolarized spectra were fit using a matching pursuit algorithm, and apparent kinetic rate constants for the exchange of pyruvate to lactate (*k*_PL_) were calculated by solving the differential form of the modified Bloch equations in the time and frequency domains.^24^ Furthermore, spectra were summed in the complex domain over time, and [1‐^13^C]lactate:[1‐^13^C]pyruvate and [^13^C]bicarbonate:[1‐^13^C]pyruvate ratios were calculated. Ratiometric and kinetic data were averaged over all subjects.

### 2.9 Statistical analysis

Statistical significance was defined as *P* < .05. The mean center frequency difference between ^13^C‐urea and ^23^Na/^1^H at site A was determined by assessing the difference in center frequency between each paired ^13^C and ^23^Na and the ^1^H measurement and averaging the results. Using in vivo data from site A, the ratiometric frequency difference between hyperpolarized ^13^C‐pyruvate and ^1^H/^23^Na across all porcine experiments was averaged.

Differences in RF gain between different saline loading states were assessed by fitting a linear model to the RF gain data.

Due to the small number of subjects used in this study, differences between time and frequency domain‐derived kinetic parameters for the in vivo data were assessed using a Mann‐Whitney U test.

## 3 RESULTS

### 3.1 RF gain and *f*_0_ estimation using ^23^Na

**FIGURE 2:**
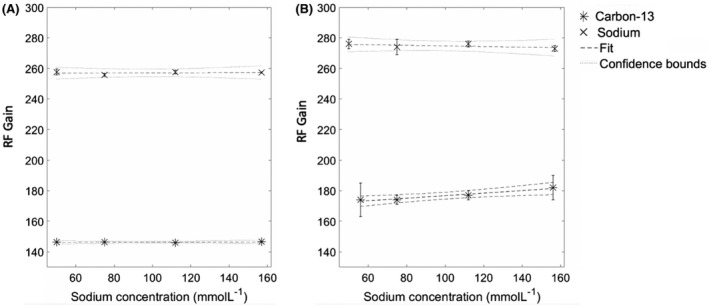
Differences in carbon‐13 and sodium‐23 RF gain required for a 90‐degree excitation at varying sodium concentrations as measured with the loop (A) and paddle (B) coils using a Bloch‐Siegert shift. The linear fit to the data and 95% confidence limits are shown. No significant correlations were found between the different loading states (R^2^ < 0.1 and *P* > .05 for all cases)

The difference in ^23^Na (NaCl) and ^13^C (^13^C‐urea) *f*_0_ at site A was 1664497 ± 12 Hz (mean ± standard deviation [SD]) with a mean scaling factor of 1.05180 ± 0.00001, as derived from Equation 1. The mean correction for the ^23^Na and ^13^C RF gain was 10.4 ± 0.6 dB, as derived from Equation 2. The correlation between saline loading and RF gain, assessed using a linear model, found no significant results for any of the coil setups (R^2^ < 0.1 and *P* > .05 in all cases; loop and paddle coil results shown in Figure 2A,B, respectively). Because no significant correlation between saline loading and RF gain was observed, data were averaged to estimate a mean RF gain difference between ^13^C and ^23^Na per coil experiment. Inspection of the calibration files for each coil demonstrated that there was a 1.35 dB difference in the additional requested system RF gain for the ^13^C loop and 8‐channel paddles. The average RF gain for each coil setup is shown in Table 1, with expanded results in Tables 2 and 3.

**Table 1:** Average results from RF gain over all saline loading conditions, per coil

Coil	^13^C RF gain	^23^Na RF gain
Loop	14.7 ± 0.1	25.7 ± 0.1
Paddles	16.4 ± 0.1	27.4 ± 0.3

Results presented as mean ± SD.

**Table 2:** TG for all saline loading conditions for both the paddle coil

Coil	Phantom Concentration	Nucleus	RF gain (dB)	SD (dB)
Paddles	156 mmolL^−^^1^	23	27.3	0.2
		13	17.4	1.1
	112 mmolL^−1^	23	27.6	0.2
		13	17.4	0.3
	75 mmolL^−1^	23	27.4	0.5
		13	17.7	0.3
	50 mmolL^−1^	23	27.6	0.3
		13	18.2	0.8

Results presented mean ± SD.

**Table 3:** TG for all saline loading conditions for the loop coil

Coil	Phantom Concentration	Nucleus	RF gain (dB)	SD (dB)
Loop	156 mmolL^−1^	23	25.7	0.1
		13	14.7	0.1
	112 mmolL^−1^	23	25.8	0.2
		13	14.6	0.1
	75 mmolL^−1^	23	25.6	0.1
		13	14.6	0.1
	50 mmolL^−1^	23	25.8	0.2
		13	14.7	0.1

Results presented mean ± SD.

### 3.2 Multinuclear *B*_1_ and *B*_0_ mapping

**FIGURE 3:**
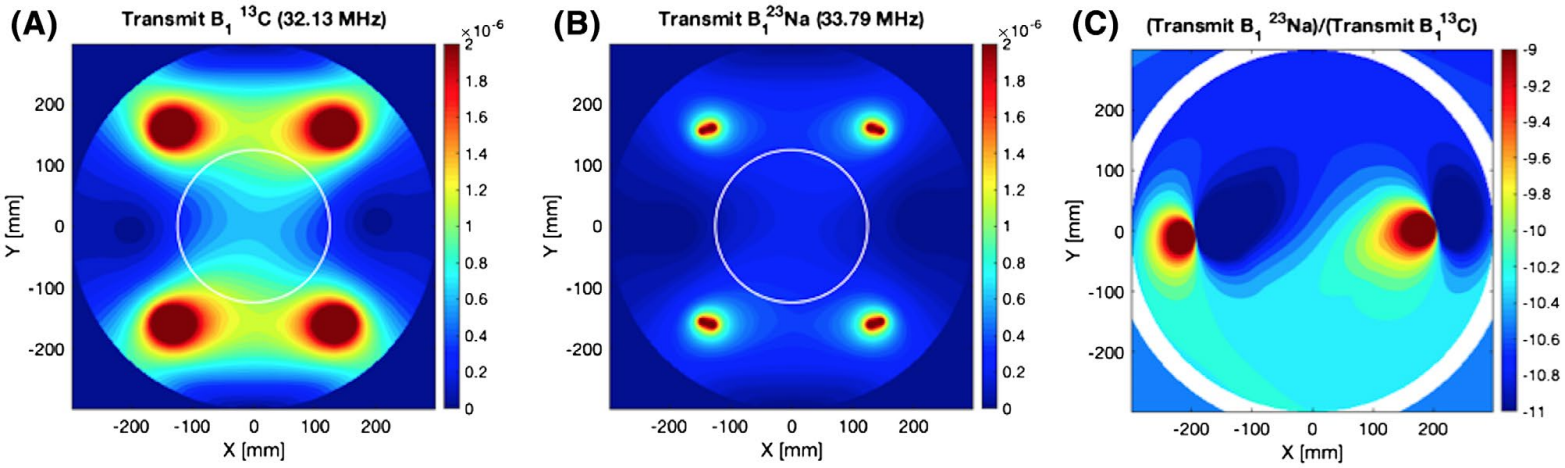
Simulated transmit *B*_1_ field distributions for the transmit volume coil used in this study. Transmit *B*_1_ distribution at ^13^C (32.13 MHz) and ^23^Na (33.79 MHz) frequencies (A and B, respectively). (C) Ratio between required power for 90‐degree flip for ^13^C and ^23^Na frequencies for the same coil. The phantom position is highlighted with a white line

**FIGURE 4:**
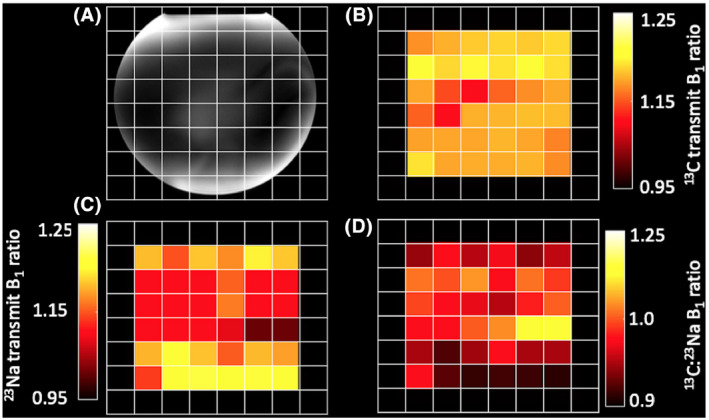
(A) Chemical shift image grid overlaid on an 8 L phantom containing 150 mmolL^−^^1^ NaCl and 102 mmolL^−1 13^C‐pyruvate. (B and C) ^13^C and ^23^Na transmit *B*_1_ maps, respectively. (D) Ratiometric difference maps between B and C

^13^C and ^23^Na *B*_0_ maps showed a mean shift of +1 ± 18 Hz and −6 ± 23 Hz over the 6 × 6 CSI grid, respectively. ^13^C and ^23^Na *B*_1_ maps showed a similar RF distribution over the FOV (Figure 3A‐C). *B*_1_ maps showed a consistent small overflipping at both ^23^Na and ^13^C frequencies (1.14 ± 0.06 and 1.18 ± 0.05, respectively). The absolute average difference between the *B*_1_ maps (Figure 4D) was 2 ± 6% over the FOV. Proton and original flip angle images can be seen in Supporting Information Figure S2.

### 3.3 Transmit *B*_1_ simulations

The simulated transmit *B*_1_ profiles are shown in Figure 4A,B for the ^13^C and ^23^Na frequencies, respectively. The difference between the field maps calculated from Equation 5 is shown in Figure 4C, demonstrating that the field distributions are very similar for both frequencies, with the maximum difference throughout the phantom being less than 2 dB. In absolute terms, the difference between the ^13^C and ^23^Na simulated field over the region of interest is 10.4 ± 0.2 dB, consistent with the experimental results.

### 3.4 Multichannel coil noise correlation

**FIGURE 5:**
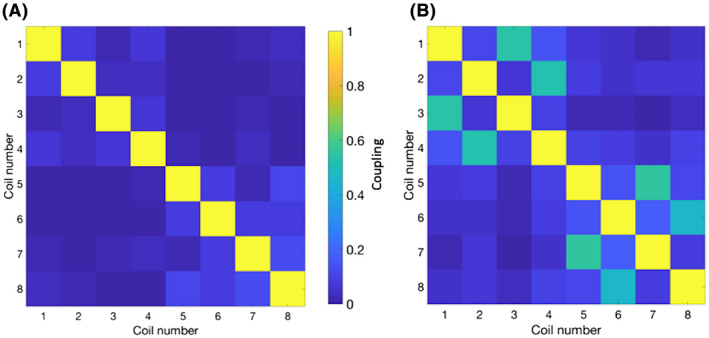
Coupling of coil channels for 8‐channel paddle coils at the carbon‐13 (A) and sodium‐23 (B) frequencies demonstrating increased coupling for the ^23^Na frequency

Noise correlation between the 8 channels in the paddle coil revealed good linearity between coils, with little correlation between channels from the off‐diagonal elements (Figure 5A). However, when performing at the ^23^Na frequency, there was substantial off‐diagonal coupling between elements (>0.6), as demonstrated in Figure 5B.

### 3.5 System magnetic field drift and ^1^H center frequency estimation from in man studies

#### 3.5.1 Temporal drift method

**FIGURE 6:**
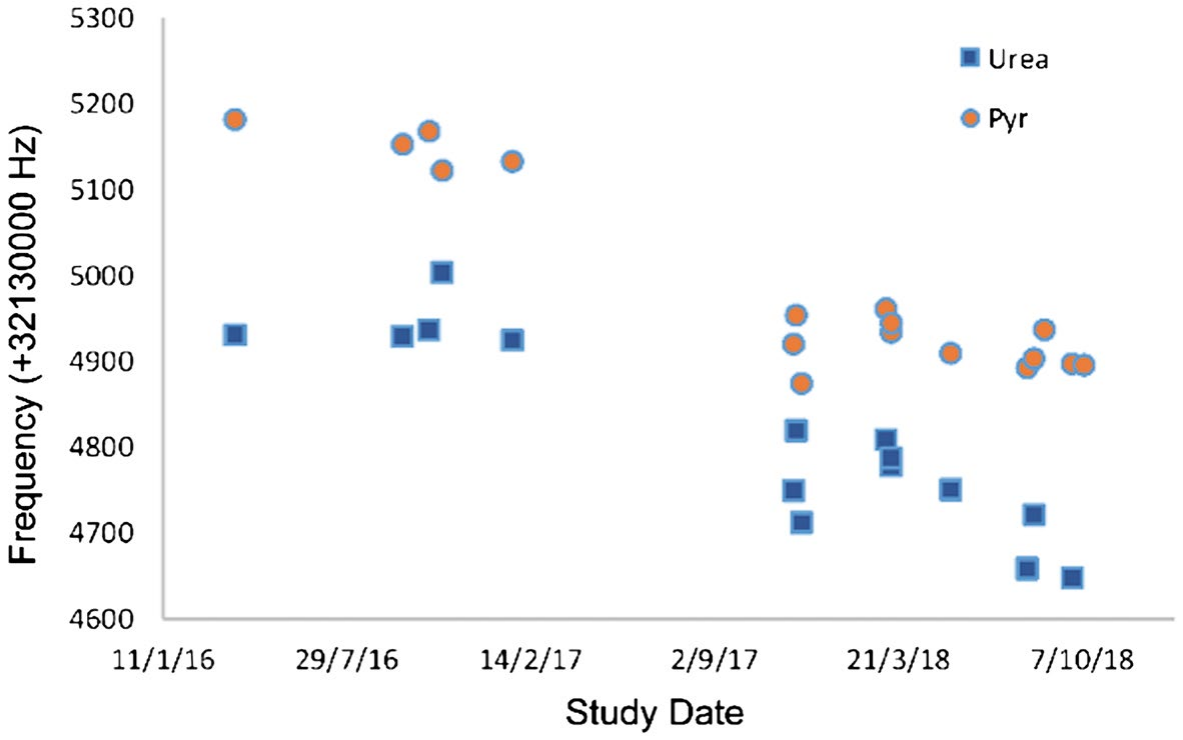
Measured frequency (+32130000 Hz) of ^13^C‐pyruvate (pyruvate) in vivo and ^13^C‐urea in vitro for all human brain hyperpolarization studies at site B over a 31‐month period

The site B frequencies of both pyruvate in vivo and urea in the phantom drifted slowly downward over the 31‐month timescale of the experiments (Figure 6). The rate of drift calculated from a linear fit to the pyruvate frequencies was 0.36 Hz/day. This drift is small in comparison to the scanner specification limits, which allow for drifts of up to 0.1 ppm/h, although such large drifts would only be expected in the case of heating due to running sequences with a high gradient duty cycle. An upgrade to the scanner software but using the existing hardware occurred in December 2017; however, this induced no discernible step change in frequency. Frequency shifts of other metabolites in vivo relative to pyruvate appeared very consistent.

#### 3.5.2 Phantom method

The difference between the frequencies of ^13^C‐pyruvate in vivo and ^13^C‐urea in the phantom varied over a wide range (186 ± 43 Hz). The frequency separation was not correlated with date of acquisition (R^2^ = 0.05), suggesting it was unlikely to be due to phantom degradation.

#### 3.5.3 Water ^1^H method

The ^1^H frequency of water scaled to the ^13^C frequency by the respective gyromagnetic ratios, as described above, was on average 2620 ± 11 Hz higher than the pyruvate frequency in vivo.

**FIGURE 7:**
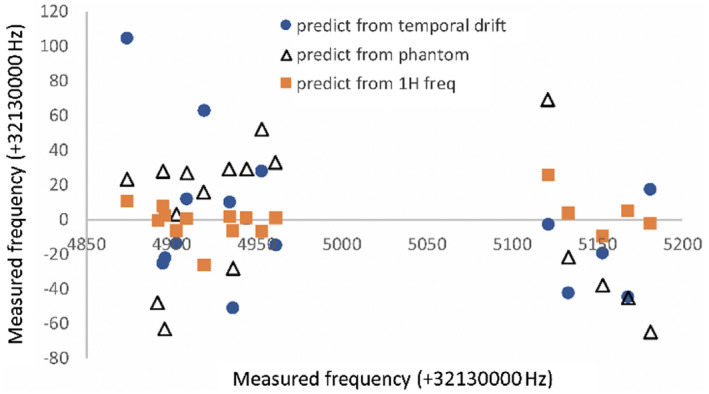
Difference between the predicted and measured frequency (+32130000 Hz) of ^13^C‐pyruvate in vivo from human brain ^13^C‐MRI studies at site B using the 3 approaches presented here. Circles: ^13^C frequency prediction from a linear fit to the study date, accounting for a drift of 0.36 Hz/day. Triangles: ^13^C frequency prediction by adding the mean offset between phantom and pyruvate to the phantom frequency. Squares: ^13^C frequency prediction based on the ^1^H frequency of water acquired from the anatomic series

Frequency prediction using the temporal drift or by referencing to the external phantom both performed poorly (Figure 7): the SD of differences (predicted minus actual) was 39 and 40 Hz, respectively, and 5 or 6 subjects, respectively, would have been outside the bandwidth of spectral–spatial excitation (error > 40 Hz) in the cohort of 17. Frequency prediction based on the water ^1^H frequency performed better, with a SD of only 11 Hz, and there were no subjects in whom spectral–spatial excitation would have failed. All results are shown in Figure 7.

### 3.6 In vivo experiments

Prospectively utilizing the ^23^Na and ^1^H prescan, hyperpolarized ^13^C‐magnetic resonance spectroscopy was successfully performed from the musculature of 6 pigs following the injection of hyperpolarized ^13^C‐pyruvate signal from ^13^C‐pyruvate, ^13^C‐lactate, and ^13^C‐bicarbonate observed in all subjects. The frequency scaling between ^23^Na/^13^C and ^13^C/^1^H calculated using Equations 1 and 2 was 1.05179 ± 0.00001 (mean ± 1 SD) and 3.97630 ± 0.00001 (mean ± 1 SD), respectively. The mean ^13^C and ^23^Na RF gain was 156 ± 2 and 260 ± 3, respectively. Kinetic analysis revealed a mean *k*_PL_ of 0.007 ± 0.002 and 0.007 ± 0.002 s^−1^ in the frequency and time domains, respectively, with no significant difference between methods (*P* > .05). The correlation (R^2^) between *k*_PL_ in the frequency domain and lactate:pyruvate ratio was 0.9 with *P* = .01.

The mean [1‐^13^C]lactate:[1‐^13^C]pyruvate (LAC/PYR) and [^13^C]bicarbonate:[1‐^13^C]pyruvate (BIC/PYR) ratios were 0.15 ± 0.04 and 0.006 ± 0.004, respectively.

## 4 DISCUSSION

Hyperpolarized ^13^C‐MRI is a powerful technique to noninvasively probe tissue metabolism in many normal and diseased tissues, such as brain, heart, kidneys, and cancer.^4^, ^8^, ^25^, ^26^, ^27^ For example, increased lactate signal has been shown to correlate with more aggressive tumors, and a reduction in this lactate is seen in a wide range of tumor models following treatment.^28^, ^29^, ^30^ This work is now being translated into humans, and early clinical research has shown elevated hyperpolarized ^13^C‐lactate in prostate cancer as well as in brain and breast tumors. Clinical hyperpolarized ^13^C studies face a number of technical challenges, including accurate frequency calibration and the requirement for system inhomogeneity correction. This is particularly problematic due to the low natural abundance of ^13^C in tissue, which results in poor signal from endogenous ^13^C nuclei, and which in turn renders the determination of the center frequency and TG difficult. Therefore, new approaches are needed to increase confidence in ^13^C prescan results, estimation of RF gain, transmission of *B*_1_ distribution, and other coil sensitivity parameters.

Here, we show that the center ^13^C frequency can be accurately estimated using the water ^1^H frequency, which performed better than 2 alternative approaches that we explored: frequency drift and using an external ^13^C‐urea phantom. In addition, we have recently shown the feasibility of using a ^13^C‐tuned coil to image the distribution of ^23^Na in tissue due to the high natural abundance of the latter and the small frequency difference between the 2 nuclei.^16^ Here, we have explored whether this ^23^Na signal could also be used to improve ^13^C‐MRI acquisition. The results show that the RF gain and *f*_0_ for ^13^C can be estimated using the endogenous ^23^Na resonance acquired using dedicated, commercially available and clinical ^13^C‐coils.

There are additional benefits in utilizing the ^23^Na resonance to calibrate the ^13^C experiments: the short T_2_ and T_1_ of the nucleus allow rapid data averaging to increase signal to noise ratio and thus increase the confidence in the calibration parameters. Conversely, the long T_1_ of ^13^C‐labeled molecules (on the order of several s) can lead to lengthy acquisition times for calibration, which further compound the problems of the low natural abundance of the nucleus.

Here, we have demonstrated the potential for using the ^1^H and ^23^Na resonances to provide a robust prescan for hyperpolarized ^13^C experiments, with center frequency and RF gain, for single and multichannel clinical coils. Simulations showed consistent overflipping at ^13^C frequency by the clamshell, which was also experimentally observed. The simulated field ^23^Na frequency was highly homogeneous, which was also experimentally observed in the *B*_1_ maps. Therefore, this provides a commercially available solution to the prescan and calibration required for ^13^C‐MRI and could assist in simplifying hyperpolarized ^13^C‐MRI acquisition. In the future, multislice *B*_1_ maps with ultrashort echo time sequences could be acquired to complement current human imaging protocols, providing a slice‐by‐slice correction for transmit *B*_1_ for further quantitation. However, this will require further experimental optimization (acquisition of coil sensitivity maps, optimal trajectory design, and eddy current compensation) before clinical implementation is achieved.^31^, ^32^ Indeed, bolus tracking methods have been developed to calibrate using the hyperpolarized signal, providing real‐time calibration for selective excitation experiments.^33^ These techniques are promising and provide an alternative approach to the method presented here. A challenge for the implementation of these methods in vivo is that they commonly require significant scanner modifications to allow for third‐party software control of the clinical system.^34^ Given that our results have also shown significant changes in acquisition parameters over time, leading to temporal alterations in the center frequency for ^1^H and ^13^C‐pyruvate, it is advisable to utilize the ^23^Na or ^1^H prescan before each experiment rather than assuming a static center frequency because selective excitation strategies may otherwise fail.

A challenge to our proposed method is the coupling between multichannel ^13^C coils, which significantly increased at the ^23^Na frequency. This could represent a problem for estimation of the sensitivity maps using array coils, but future ^13^C coil design could ensure that this effect is minimized. Future studies could assess this concept in human experiments, including a comparison of *k*_PL_ values before and after correction with a sodium *B*_1_ map. Uncertainty in transmit *B*_1_ dramatically increases the error in kinetic modeling, which could have significant implications for the interpretation of the results as part of clinical trials.^10^ Interestingly, there appeared to be a difference in the RF gain required for the 8‐channel paddle coils and the single loop coil used in this study, with an approximate difference of 2 dB. This difference is partially due to the difference in vendor coil files (1.35 dB), and we hypothesize that the remainder is due to differences in coil positioning and volumetric coverage because the transmit clamshell has a nonuniform transmit field (Figure 4). Further differences in TG could be found in variations in coil matching at the ^23^Na frequency, leading to differences in TG required for a 90‐degree excitation. Reductions in transmit efficiency are expected when operating away from the intended frequency due to degradation in the quality of the tune and match of the coil, although the relatively small difference in TG needed here suggests this degradation is not severe. In the future, the TG could also be used to indirectly inform on the quality of the tune and match. Although we have demonstrated here that ^13^C coils can be successfully used to measure the ^23^Na resonance, this approach should be applied with caution by assessing the coil response at both frequencies on the bench and also by undertaking studies in phantoms prior to performing these studies in humans.^35^

## 5 CONCLUSION

We have demonstrated the feasibility of using the ^23^Na and ^1^H resonances to calibrate the ^13^C transmit *B*_1_ using commercially available ^13^C‐coils. The method provides a simple approach for in vivo calibration and could improve clinical workflow.

## CONFLICT OF INTEREST

Dr Rolf Schulte is an employee of GE Healthcare.

## Supporting information


**FIGURE S1** Phantom setup. Saline filled buckets (150 mmolL^−1^, 1 L) with the 8‐channel paddle coils (A) or the single loop coil (B) inside the clamshell transmit coil. The black arrow points to the ^13^C enriched urea phantom
**FIGURE S2** Individual ^1^H (A) and x‐nuclei flip angle images for ^23^Na (B and C, 40 and 80 degrees, respectively) and ^13^C (D and E, 40 and 80 degrees, respectively) acquired from an 8 L phantom. ^23^Na and ^13^C images are normalized to the largest signal in their 80‐degree images
