# Supplementary material for: Creating a clinical platform for carbon‐13 studies using the sodium‐23 and proton resonances
Source: Magn Reson Med. 2020 Mar 13;84(4):1817–27. doi: 10.1002/mrm.28238 (PMC8638662; doi:10.1002/mrm.28238)
Supplement: Supplementary file 1 — FIGURE S1 Phantom setup. Saline filled buckets (150 mmolL−1, 1 L) with the 8‐channel paddle coils (A) or the single loop coil (B) inside the clamshell transmit coil. The black arrow points to the 13C enriched urea phantom FIGURE S2 Individual 1H (A) and x‐nuclei flip angle images for 23Na (B and C, 40 and 80 degrees, respectively) and 13C (D and E, 40 and 80 degrees, respectively) acquired from an 8 L phantom. 23Na and 13C images are normalized to the largest signal in their 80‐degree images [file MRM-84-1817-s001.docx]

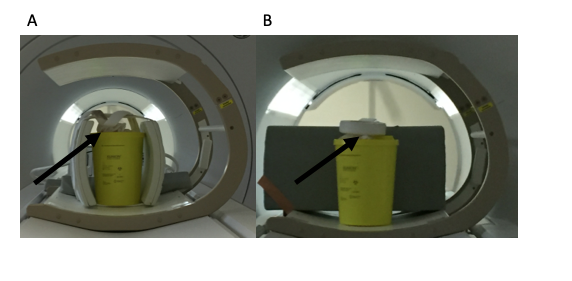


**Supporting Information Figure S1:** Phantom set-up. Saline filled buckets (150 mmolL^-1^, 1L) with the 8-channel paddle coils (A) or the single loop coil (B) inside the clamshell transmit coil. The black arrow points to the ^13^C enriched Urea phantom.


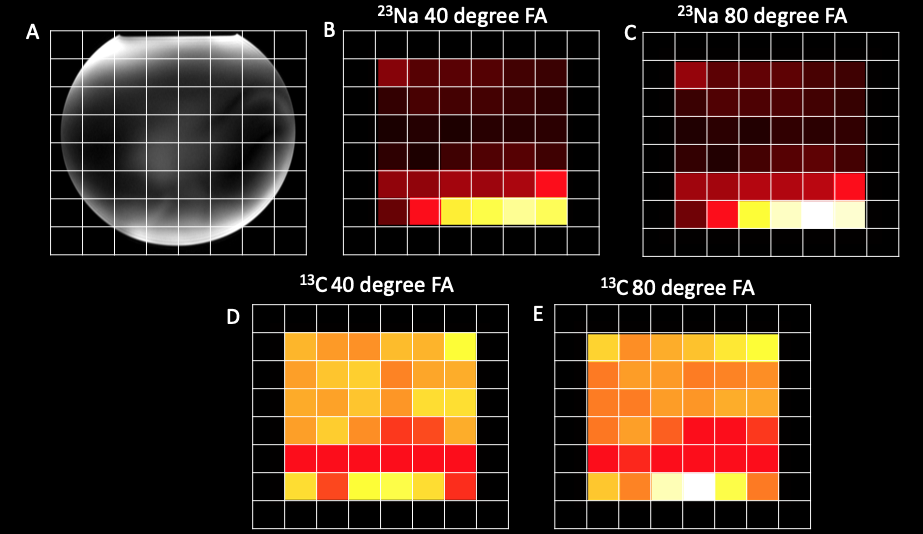


**Supporting Information Figure S2:** Individual ^1^H (A) and x-nuclei flip angle images for ^23^Na (B and C, 40 and 80 degrees, respectively) and ^13^C (D and E, 40 and 80 degrees, respectively). ^23^Na and ^13^C images are normalized to the largest signal in their 80-degree images.
